# Supplementary material for: Transcranial magnetic stimulation enhances the specificity of multiple sclerosis diagnostic criteria: a critical narrative review
Source: PeerJ. 2024 Mar 29;12:e17155. doi: 10.7717/peerj.17155 (PMC10984191; doi:10.7717/peerj.17155)
Supplement: Supplemental Information 1 [file peerj-12-17155-s001.docx]

**Transcranial magnetic stimulation enhances the specificity of multiple sclerosis diagnostic criteria: A critical narrative review.**

Nicholas J. Snow, Hannah M. Murphy, Arthur R. Chaves, Craig S. Moore, & Michelle Ploughman

# **Supplementary Materials**

1. Review protocol.
2. **Table S1.** Study characteristics.
3. **Table S2.** Control participant characteristics.
4. **Table S3.** Transcranial magnetic stimulation characteristics.
5. **Table S4.** Detailed transcranial magnetic stimulation results.
6. **Table S5.** Detailed risk of bias assessment.
7. **Table S6.** Detailed biomarker assessment.
8. References.
9. **Table S7.** Document tracking spreadsheet (separate document).
10. **Table S8.** Data transcription spreadsheet (separate document).

**Review Protocol**

**Eligibility Criteria**

- Journal articles,
- English language,
- Original research,
- Not pediatric sample,
- Humans,
- CIS → MS, HC vs. MS,
- MS patient sample size, *n* ≥ 40,
- Validated diagnostic criteria,
- Diagnostic classification data (sensitivity and/or specificity),
- No case reports,
- TMS-EMG of upper or lower extremities,
- No combined evoked potentials scores.

**Search Date**

February 28, 2022

**PubMED**

Terms

("multiple sclerosis"[all fields] OR "clinically isolated syndrome"[all fields]) AND ("transcranial magnetic stimulation"[all fields]) AND (sensitiv*[all fields] OR specific*[all fields] OR "predictive value"[all fields] OR "likelihood ratio"[all fields] OR "odds ratio"[all fields] OR "risk ratio"[all fields] OR "hazard ratio"[all fields])

Results

50

**Embase**

Terms

('multiple sclerosis'/exp OR 'multiple sclerosis' OR 'clinically isolated syndrome'/exp OR 'clinically isolated syndrome') AND ('transcranial magnetic stimulation'/exp OR 'transcranial magnetic stimulation') AND (sensitiv* OR specific* OR 'predictive value'/exp OR 'predictive value' OR 'likelihood ratio'/exp OR 'likelihood ratio' OR 'odds ratio'/exp OR 'odds ratio' OR 'risk ratio'/exp OR 'risk ratio' OR 'hazard ratio'/exp OR 'hazard ratio')

Results

121

**Web of Science**

Terms

(ALL=("multiple sclerosis") OR ALL=("clinically isolated syndrome")) AND (ALL=("transcranial magnetic stimulation") AND (ALL=(sensitiv*) OR ALL=(specific*) OR ALL=("predictive value") OR ALL=("likelihood ratio") OR ALL=("odds ratio") OR ALL=("risk ratio") OR ALL=("hazard ratio"))

Results

90

**Scopus**

Terms

ALL ( ( "multiple sclerosis" [all AND fields] OR "clinically isolated syndrome" [all AND fields] ) AND ( "transcranial magnetic stimulation" [all AND fields] ) AND ( sensitiv*[all AND fields] OR specific*[all AND fields] OR "predictive value" [all AND fields] OR "likelihood ratio" [all AND fields] OR "odds ratio" [all AND fields] OR "risk ratio" [all AND fields] OR "hazard ratio" [all AND fields] ) )

Results

676

**Article Inclusion**

Total from Searches

937

Reviews Flagged

12

Total from Reviews

85

Total from Included Items

51

**Total from All Sources**

1073

Duplicates

109

**For Title Review**

964

For Abstract Review

268

For Full Text Review

201

**Final**

17

**Table S1.** Study characteristics.

| **Study** | **Funding source** | **Prospective study** | **Consecutive patients** | **Longitudinal observation** | **Examines subclinical lesions** | **RMS vs. PrMS** | **Low vs. high disability** | **Active vs. inactive MS** |
| --- | --- | --- | --- | --- | --- | --- | --- | --- |
| (Beer et al. 1995) | Swiss National Science Foundation [Grant No 3.852-1.86] | Y | Y | N | Y | N | N | N |
| (Caramia et al. 2004) | NR | Y | N | Y | N | N | N | Y |
| (Cruz-Martínez et al. 2000) | NR | Y | Y | Y | Y | N | N | N |
| (Facchetti et al. 1997) | NR | N | Y | N | N | Y | N | N |
| (Hess et al. 1987) | UK Multiple Sclerosis Society,  Roche Research Foundation,  Rotary Foundation | N | Y | N | Y | N | N | N |
| (Jung et al. 2006) | Biogen Idec. | N | N | N | Y | N | N | N |
| (Kale et al. 2009) | NR | N | Y | N | Y | N | Y | N |
| (Kale et al. 2010) | NR | N | N | N | N | N | Y | N |
| (Kandler et al. 1991) | NR | N | Y | N | Y | N | N | N |
| (Leocani et al. 2006) | Italian National Ministry of Health [Grant No 96/J/T44] | Y | Y | Y | N | Y | N | N |
| (Magistris et al. 1999) | Swiss National Science Foundation [Grant No 31–43454.95] | Y | Y | N | Y | N | N | N |
| (Mayr et al. 1991) | NR | N | N | N | Y | N | N | N |
| (Pisa et al. 2020) | Fondazione Italiana Sclerosi Multipla [Grant No FISM2012/R/9] | N | Y | N | N | N | N | N |
| (Ravnborg et al. 1992) | Danish Multiple Sclerosis Society, Fondsborsvekselerer Henry Hansen Og Hustru,  Carla Hansen,  Fodt Westergaards Legat, Lykfeldts Legat, Foundation for Experimental Research in Neurology | Y | Y | N | Y | N | N | N |
| (Schmierer et al. 2000) | Charité Forschungsförderung [Grant No 97–209] | N | N | N | N | N | N | N |
| (Schmierer et al. 2002) | NR | Y | N | Y | N | Y | Y | N |
| (Tataroglu et al. 2003) | NR | N | Y | N | Y | Y | N | N |

MS, multiple sclerosis; N, no; NR, not reported; PrMS, progressive multiple sclerosis; RMS, relapsing multiple sclerosis; Y, yes.

**Table S2.** Control participant characteristics.

| **Study** | **Control group** | **Other neurologic disorders** | **Sample size** | **# Females** | **Age** | **Age-matched to MS** | **Sex-matched to MS** |
| --- | --- | --- | --- | --- | --- | --- | --- |
| (Beer et al. 1995) | Other neurologic disorders | Functional neurologic symptoms, Stroke, Non inflammatory myelopathy, Other neurologic disorders | 47 | 27 | 41 (19-67) | NR | NR |
| (Caramia et al. 2004) | Healthy controls | NA | 20 | 12 | 36.2 (18-52) | Y | NR |
| (Cruz-Martínez et al. 2000) | Healthy controls | NA | 38 | 23 | 32.7 (14-56) | Y | NR |
| (Facchetti et al. 1997) | Healthy controls | NA | 20 | 10 | 30.1 ± 5.5 | NR | NR |
| (Hess et al. 1987) | Healthy controls | NA | 32 | 5 | 36.8 (21-78) | NR | NR |
| (Jung et al. 2006) | Healthy controls | NA | 20 | 7 | 29 (18-44) | NR | NR |
| (Kale et al. 2009) | Healthy controls | NA | 53 | 35 | 39 ± 9.5 | NR | NR |
| (Kale et al. 2010) | Healthy controls | NA | 50 | 31 | 35.4 ± 10.4 | Y | Y |
| (Kandler et al. 1991) | Healthy controls | NA | 30 | 12 | 38 (22-74) | NR | NR |
| (Leocani et al. 2006) | Healthy controls | NA | NR | NR | NR | NR | NR |
| (Magistris et al. 1999) | Other neurologic disorders | Stroke, Neurodegenerative disorders, ALS, Myelopathy, Peripheral nerve disorders, Functional neurologic symptoms, Other neurologic disorders,  Non neurologic disorders | 155 | NR | 51 (17-90) | NR | NR |
| (Mayr et al. 1991) | Healthy controls | NA | 86 | 49 | 38.2 (17-72) | NR | NR |
| (Pisa et al. 2020) | Healthy controls | NA | 10 | NR | NR | Y | Y |
| (Ravnborg et al. 1992) | Healthy controls | NA | 50 | 25 | 18-60 | NR | NR |
| (Schmierer et al. 2000) | Healthy controls | NA | 25 | 14 | 31.8 (23-46) | NR | NR |
| (Schmierer et al. 2002) | Healthy controls | NA | 35 | 18 | 36 (20-62) | Y | Y |
| (Tataroglu et al. 2003) | Healthy controls | NA | 31 | 18 | 33.4 (22-49) | NR | NR |

Note: age and disease duration are reported in years. Continuous data are expressed as median (range) or mean ± standard deviation. ALS, amyotrophic lateral sclerosis; MS, multiple sclerosis; N, no; NA, not applicable; NR, not reported; Y, yes.

**Table S3.** Transcranial magnetic stimulation characteristics.

| **Study** | **Handedness** | **CNS drugs** | **TMS measure** | **MT** | **Target** | **Side** | **Electrodes** | **Contraction of target muscle** | **Muscle activity monitoring** | **Type of stimulator** | **Pulse shape** | **Coil type** | **Coil orientation** | **Current direction** | **Coil location** | **Hotspot** | **Intensity** | **Attention** | **# Trials** | **Inter-trial interval** | **MEP size** | **Room temperature** |
| --- | --- | --- | --- | --- | --- | --- | --- | --- | --- | --- | --- | --- | --- | --- | --- | --- | --- | --- | --- | --- | --- | --- |
| (Beer et al. 1995) | NR | NR | CMCT (NR) | RMT | ADM, BB, TA | Both | Bipolar | Relaxed | EMG monitored | Custom | Monophasic | 9 cm C | Tangential to scalp | Clockwise, Anticlockwise | Marked using pen | Motor mapping (2 cm steps) | > RMT | NR | NR | NR | NR | NR |
| (Caramia et al. 2004) | MS: 7 Lt weak, 16 Rt weak, 2 both weak, 54 not weak Control: NR | No | CMCT (F-wave) | RMT | OPB | More clinically affected | Bipolar | 40% of maximum | Auditory EMG feedback | Magstim 200 | Monophasic | 9.5 cm F8 | Tangential to scalp, handle 45^o^ to sagittal plane | Posterior-anterior | Over hotspot | NR | 105% RMT | EMG feedback | 3 | 7 s | NR | NR |
| (Cruz-Martínez et al. 2000) | NR | NR | RMT, MEP (Amplitude), CMCT (F-wave) | RMT | OPB, TA | Both | Bipolar | Slight | NR | Magstim 200 | Monophasic | C | Tangential to scalp | Clockwise, Anticlockwise | Over vertex or just anterior | NR | 130% RMT | NR | 6-10 | NR | Normalized to CMAP | NR |
| (Facchetti et al. 1997) | NR | NR | CMCT (Nerve root) | RMT | ADM, TA | Both | Bipolar | Slight | NR | Cadwell MS10 | Monophasic | NR | NR | NR | NR | NR | 130% RMT | NR | 4 | NR | NA | NR |
| (Hess et al. 1987) | NR | NR | MEP (Amplitude), CMCT (Nerve root) | AMT | ADM | Both | Bipolar | 5-10% of maximum | EMG monitored | Custom | Monophasic | 9 cm C | Tangential to scalp | Clockwise, Anticlockwise | Over vertex | NR | > AMT | NR | 4 | NR | Normalized to CMAP | NR |
| (Jung et al. 2006) | NR | NR | CMCT (Nerve root), iSP ([automated] Latency, duration, depth, TCT [iSP-MEP]) | RMT | APB, TA | Both | Bipolar | Maximal | Auditory EMG feedback | Magstim 200 | Monophasic | 9 cm F8 | Tangential to scalp, handle 0-45° from sagittal plane | Posterior-anterior | Over hotspot | Location of reproducible MEPs | 80% MSO | EMG feedback | 15 | 5-7 s | iSP normalized to pre-stimulus EMG | NR |
| (Kale et al. 2009) | NR | NR | MEP (Amplitude, latency), CMCT (Nerve root) | RMT | APB | Both | Bipolar | Relaxed | EMG monitored | Magstim 200 | Monophasic | C | Tangential to scalp | Clockwise, Anticlockwise | Over hotspot | Location of reproducible MEPs | NR | NR | 5 | NR | Amplitude of raw MEP | Air-conditioned |
| (Kale et al. 2010) | NR | NR | MEP (Area, latency), CMCT (Nerve root) | RMT | APB | Both | Bipolar | Relaxed | EMG monitored | Magstim 200 | Monophasic | C | Tangential to scalp | Clockwise, Anticlockwise | Over hotspot | Location of reproducible MEPs | NR | NR | 5 | NR | Raw MEP | Air-conditioned |
| (Kandler et al. 1991) | NR | NR | MEP (Amplitude), CMCT (Nerve root) | NR | ADM, AH | Both | Bipolar | Relaxed | Auditory EMG feedback | Magstim 200 | Monophasic | C | Tangential to scalp | Clockwise, Anticlockwise | Over vertex or just anterior | NR | 90-100% MSO | EMG feedback | 6 | NR | Raw MEP | NR |
| (Leocani et al. 2006) | NR | NR | CMCT (Nerve root) | NR | APB, AH | Both | NR | 20% of maximum | NR | Cadwell MS10 | Monophasic | 12 cm C | Tangential to scalp | Clockwise, Anticlockwise | Over vertex | NR | NR | NR | NR | NR | NR | NR |
| (Magistris et al. 1999) | NR | NR | CMCT (F-wave), TST (Amplitude, area) | AMT | ADM | Both | Bipolar | Slight | Auditory EMG feedback | Magstim 200 | Monophasic | 9 cm C | Tangential to scalp | Clockwise, Anticlockwise | Over vertex or just lateral | Location of lowest AMT | NR | EMG feedback | 8 | NR | TST normalized to control curve | NR |
| (Mayr et al. 1991) | NR | NR | MEP (Amplitude), CMCT (F-wave) | RMT | OPB, AH | Both | Bipolar | Slight | Auditory EMG feedback | Magstim 200 | Monophasic | 8.5 cm C | Tangential to scalp | Clockwise, Anticlockwise | Over vertex or 1-2 cm anterior | NR | 120% RMT | EMG feedback | 4 | NR | Raw MEP | NR |
| (Pisa et al. 2020) | MS: 9 unilaterally weak, 11 both weak, 17 not weak, 13 NR, Control: NR | NR | MEP (Latency) | RMT | FDI, TA | Dominant | Bipolar | Relaxed | NR | Magstim 200 | Monophasic | 7 cm F8 | Tangential to scalp | NR | Marked with pen | NR | 120% RMT | NR | 10 | NR | NR | NR |
| (Ravnborg et al. 1992) | NR | NR | MEP (Amplitude), CMCT (Nerve root) | AMT | BB, FCR, FDI, TA, AH | Both | Bipolar | 10-15% of maximum | Visual EMG feedback | Dantec | Monophasic | 14 cm C | Tangential to scalp, handle in sagittal plane | Clockwise, Anticlockwise | 1 cm anterior to vertex | NR | 120% AMT | EMG feedback | 3 | NR | Normalized to spine root MEP | 22^o^C |
| (Schmierer et al. 2000) | NR | NR | MEP (Amplitude), CMCT (Nerve root), iSP ([visual] latency, duration, TCT [iSP-MEP]) | AMT | FDI, TA | Both | Bipolar | Maximal | EMG monitored | Magstim 200 | Monophasic | 8.5 cm F8 | Tangential to scalp, handle anterior-posterior | Posterior-anterior | Over hotspot | Location of maximal MEP | 80% MSO | NR | 20 | 2-3 s | Raw MEP | NR |
| (Schmierer et al. 2002) | NR | NR | RMT (Relative frequency), CMCT (Nerve root), iSP ([visual] latency, duration, TCT [iSP-MEP]) | RMT | FDI, TA | Both | Bipolar | Maximal | EMG monitored | Magstim 200 | Monophasic | 8.5 cm F8 | Tangential to scalp, with handle anterior-posterior | Posterior-anterior | Over hotspot | Location of maximal MEP | 80% MSO | NR | 20 | 3 s | NA | NR |
| (Tataroglu et al. 2003) | NR | NR | MEP (Amplitude, latency) CMCT (Nerve root), CSP (Duration, latency; method)* | RMT | FDI, TA | Both | NR | 50% of maximum | Visual EMG feedback | Magstim 200 | Monophasic | 9 cm C | Tangential to scalp | Clockwise, Anticlockwise | Over vertex or 2-3 cm anterior | NR | 150% RMT | EMG feedback | 5 | NR | Normalized to CMAP | NR |

Note: no study reported history of repetitive motor activity or level of relaxation of non-target muscles; thus, these criteria are omitted from the table. ADM, abductor digiti minimi; AH, abductor hallucis; AMT, active motor threshold; APB, abductor pollicis brevis; BB, biceps brachii; C, circular; CMAP, compound muscle action potential; CMCT, central motor conduction time; CNS, central nervous system; CSP, corticospinal silent period; Delt, deltoid; EMG, electromyography; F8, figure-of-eight; FCR, flexor carpi radialis; FDI, first dorsal interosseus; iSP, ipsilateral silent period; Lt, left; MEP, motor evoked potential; MSO, maximum stimulator output; NR, not reported; OPB, opponens pollicis brevis; RMT, resting motor threshold; Rt, right; TA, tibialis anterior; TCT, transcallosal conduction time; TST, triple stimulation technique. *, method for CSP determination not reported. See ref: (Chipchase et al. 2012).

**Table S4.** Detailed transcranial magnetic stimulation results.

| **Study** | **Abnormal result criterion** | **2 × 2 contingency result** | **Sensitivity (95% CI)** | **Specificity (95% CI)** | **Diagnostic odds ratio (95% CI)** | **Associations with disease-related outcomes** |
| --- | --- | --- | --- | --- | --- | --- |
| ***Resting motor threshold (RMT), one study (6%)*** | | | | | | |
| (Cruz-Martínez et al. 2000) | > 2 SD above mean of controls | **Upper extremity:** TP = 34/88 limbs, FP = 0/38 participants, TN = 38/38 participants, FN = 54/88 limbs  **Lower extremity:** TP = 15/35 limbs, FP = 0/38 participants, TN = 38/38 participants, FN = 20/35 limbs | **Upper extremity:** 39%  **Lower extremity:**  43% | **Upper extremity:** 100% (98-100%)  **Lower extremity:** 100% (98-100%) | **Upper extremity:** 23.30 (13.66-39.75)  **Lower extremity:** 27.75 (13.22-58.23) | RMT was correlated with EDSS (*p* < .02), ataxia (*p* < .04), and central motor pathway MRI lesions (*p* < .05).  Magnitude not reported. |
| (Schmierer et al. 2002) | > 2.5 SD above mean of controls | **RMS (upper + lower extremity):** TP = 7/38 limbs, FP = NR, TN = NR, FN = 31/38 limbs  **PPMS (upper + lower extremity):** TP = 4/38 limbs, FP = NR, TN = NR, FN = 34/38 limbs | **RMS (upper + lower extremity):** 18%  **PPMS (upper + lower extremity):** 10% | NR | NR | RMT was not significantly correlated with EDSS. |

| ***Motor evoked potential (MEP), 10 studies (59%)*** | | | | | | |
| --- | --- | --- | --- | --- | --- | --- |
| *MEP size (amplitude, area), nine studies (53%)* | | | | | | |
| (Cruz-Martínez et al. 2000) | > 2 SD below mean of controls | **Upper extremity:** TP = 21/88 limbs, FP = 0/38 participants, TN = 38/38 participants, FN = 67/88 limbs  **Lower extremity:** TP = 10/35 limbs, FP = 0/38 participants, TN = 38/38 participants, FN = 25/35 limbs | **Upper extremity:** 24%  **Lower extremity:** 29% | **Upper extremity:** 100% (98-100%)  **Lower extremity:** 100% (98-100%) | **Upper extremity:** 11.60 (6.47-20.81)  **Lower extremity:** 14.80 (6.65-32.92) | MEP amplitude was correlated with EDSS (*p* < .03), ataxia (*p* < .007), and MRI lesions in the pons (*p* < .009) and cervical cord (*p* < .03).  Magnitude not reported. |
| (Hess et al. 1987) | < 15% of CMAP amplitude | **Upper extremity:** TP = 39/83 participants, FP = 0/32 participants, TN = 32/32 participants, FN = 44/83 participants | **Upper extremity:** 47% | **Upper extremity:** 100% (94-100%) | **Upper extremity:** 27.48 (15.81-47.78) | NR |
| (Kale et al. 2009) | > 2.5 SD below mean of controls | **Upper extremity:** TP = 109/131 participants, FP = NR, TN = NR, FN = 22/131 participants | **Upper extremity:** 83% (82-84%) | NR | NR | MEP amplitude was correlated with EDSS (*p* < .001).  Magnitude not reported. |
| (Kale et al. 2010) | > 2.5 SD below mean of controls | **Upper extremity:** TP = 67/79 participants, FP = NR, TN = NR, FN = 12/79 participants | **Upper extremity:** 85% (83-87%) | NR | NR | MEP amplitude was correlated with EDSS (*p* < .05) and corpus callosum atrophy (*p* not reported).  Magnitude not reported. |
| (Kandler et al. 1991) | Below mean of controls | **Upper extremity:** TP = 14/162 limbs, FP = NR, TN = NR, FN = 148/162 limbs  **Lower extremity:** TP = 16/63 limbs, FP = NR, TN = NR, FN = 47/63 limbs | **Upper extremity:** 9%  **Lower extremity:** 25% | NR | NR | MEP amplitude was correlated with pyramidal dysfunction (hyperreflexia, weakness, spasticity, plantar reflex) (*p* not reported).  Magnitude not reported. |
| (Mayr et al. 1991) | < 1 %ile of controls | **Upper extremity:** TP = 5/44 participants, FP = 4/86 participants, TN = 82/86 participants, FN = 39/44 participants  **Lower extremity:** TP = 12/44 participants, FP = 4/86 participants, TN = 82/86 participants, FN = 32/44 participants | **Upper extremity:** 11%  **Lower extremity:** 28% | **Upper extremity:** 99% (97-100%)  **Lower extremity:** 100% (98-100%) | **Upper extremity:** 2.63 (0.67-10.34)*  **Lower extremity:** 7.69 (2.31-25.61) | MEP amplitude was not significantly correlated with pyramidal dysfunction (hyperreflexia, weakness, spasticity, plantar reflex). |
| (Ravnborg et al. 1992) | < lower 99% confidence limit of controls | **Upper + lower extremity:** TP = 20/40 participants, FP = 4/28 participants, TN = 24/28 participants, FN = 20/40 participants | **Upper + lower extremity:** 50% (38-63%) | **Upper + lower extremity:** 86% (81-100%) | **Upper + lower extremity:** 6.00 (1.76-20.46) | MEP amplitude was correlated with MRI lesion number (McNemar’s = 0.85, *p* not reported) but not pyramidal dysfunction (hyperreflexia, weakness, spasticity, plantar reflex). |
| (Schmierer et al. 2000) | Below lowest value of controls | **Upper extremity:** TP = 17/50 participants, FP = 0/25 participants, TN = 25/25 participants, FN = 33/50 participants  **Lower extremity:** TP = 3/50 participants, FP = 0/25 participants, TN = 25/25 participants, FN = 47/50 participants | **Upper extremity:** 34%  **Lower extremity:** 6% | **Upper extremity:** 100% (92-100%)  **Lower extremity:** 100% (92-100%) | **Upper extremity:** 12.36 (6.11-25.00)  **Lower extremity:** 1.53 (0.45-5.24)* | MEP amplitude was not significantly correlated with MRI lesion location or burden. |
| (Tataroglu et al. 2003) | > 2.5 SD below mean of controls,  Asymmetry > 2.5 SD above mean of controls | **Upper + lower extremity:** TP = 38/58 participants, FP = 2/31 participants, TN = 29/31 participants, FN = 20/58 participants | **Upper + lower extremity:** 66% (64-68%) | **Upper + lower extremity:** 94% (88-100%) | **Upper + lower extremity:** 27.55 (5.95-127.46) | MEP amplitude was not significantly correlated with EDSS. |
| *MEP latency, four studies (24%)* | | | | | | |
| (Kale et al. 2009) | > 2.5 SD above mean of controls | **Upper extremity:** TP = 68/131 participants, FP = NR, TN = NR, FN = 63/131 participants | **Upper extremity:** 52% (52-52%) | NR | NR | MEP latency was correlated with EDSS (*p* < .001).  Magnitude not reported. |
| (Kale et al. 2010) | > 2.5 SD above mean of controls | **Upper extremity:** TP = 34/79 participants, FP = NR, TN = NR, FN = 45/79 participants | **Upper extremity:** 43% | NR | NR | MEP latency was correlated with corpus callosum atrophy (*p* not reported) but not EDSS.  Magnitude not reported. |
| (Pisa et al. 2020) | Upper extremity > 24.3 ms,  Lower extremity > 36.5 ms | **Upper extremity:** TP = 41/50 participants, FP = NR, TN = NR, FN = 9/50 participants  **Lower extremity:** TP = 49/50 participants, FP = NR, TN = NR, FN = 1/50 participants | **Upper extremity:** 82% (79-85%)  **Lower extremity:** 98% (94-100%) | NR | NR | Upper extremity MEP latency was correlated with EDSS (Rho = 0.296, *p* < .05) and walking performance (Rho = 0.6, *p* < .0001).  Lower extremity MEP latency not reported. |
| (Tataroglu et al. 2003) | > 2.5 SD above mean of controls,  Asymmetry > 2.5 SD above mean of controls | **Upper + lower extremity:** TP = 40/58 participants, FP = 6/31 participants, TN = 25/31 participants, FN = 18/58 participants | **Upper + lower extremity:** 69% (67-71%) | **Upper + lower extremity:** 80% (75-85%) | **Upper + lower extremity:** 9.26 (3.24-26.47) | MEP latency was not significantly correlated with EDSS. |
| ***Central motor conduction time (CMCT), 16 studies (94%)*** | | | | | | |
| (Beer et al. 1995) | > 2.5 SD above mean of entire sample,  Asymmetry > 2.5 SD above mean of entire sample | **Upper + lower extremity:** TP = 96/142 participants, FP = 11/47 participants, TN = 36/47 participants, FN = 46/142 participants | **Upper + lower extremity:** 68% (67-69%) | **Upper + lower extremity:** 77% (74-80%) | **Upper + lower extremity:** 6.83 (3.19-14.62) | NR |
| (Caramia et al. 2004) | > 2 SD above mean of controls | **Upper extremity:** TP = 13/79 participants, FP = NR, TN = NR, FN = 66 participants | **Upper extremity:** 16% | NR | NR | NR |
| (Cruz-Martínez et al. 2000) | > 2 SD above mean of controls | **Upper extremity:** TP = 54/88 limbs, FP = 0/38 participants, TN = 38/38 participants, FN = 34/88 limbs  **Lower extremity:** TP = 18/35 limbs, FP = 0/38 participants, TN = 38/38 participants, FN = 17/35 limbs | **Upper extremity:** 61% (60-62%)  **Lower extremity:** 51% (50-52%) | **Upper extremity:** 100% (98-100%)  **Lower extremity:** 100% (98-100%) | **Upper extremity:** 58.76 (34.45-100.24)  **Lower extremity:** 39.17 (18.78-81.70) | CMCT was correlated with EDSS (*p* < .01), pyramidal dysfunction (hyperreflexia, weakness, spasticity, plantar reflex) (*p* < .02), ataxia (*p* < .02), and MRI lesions in the pons (*p* < .03) and central motor pathway (*p* < .04).  Magnitude not reported. |
| (Facchetti et al. 1997) | > 2.5 SD above mean of controls | **RMS (upper extremity):** TP = 12/40 participants, TN = NR, FP = NR, FN = 28/40 participants  **SPMS (upper extremity):**  TP = 13/13 participants, FP = NR, TN = NR, FN = 0/13 participants  **RMS (lower extremity):** TP = 17/40 participants, FP = NR, TN = NR, FN = 23/40 participants  **SPMS (lower extremity):** TP SPMS = 13/13 participants, FP = NR, TN = NR, FN SPMS = 0/13 participants | **RMS (upper extremity):** 30%  **SPMS (upper extremity):**  100% (85-100%)  **RMS (lower extremity):** 43%  **SPMS (lower extremity):** 100% (85-100%) | NR | NR | CMCT was not significantly correlated with EDSS or number or area of MRI lesions. |
| (Hess et al. 1987) | > 2.5 SD above mean of controls  Asymmetry > 2.5 SD above mean of controls | **Upper extremity:** TP = 60/83 participants, FP = 0/32 participants, TN = 32/32 participants, FN = 23/83 participants | **Upper extremity:** 72% (70-74%) | **Upper extremity:** 100% (94-100%) | **Upper extremity:** 80.87 (44.71-146.26) | CMCT was correlated with hyperreflexia (*p* < .001), weakness (*p* < .05), and ataxia (*p* < .05), but not impaired fine movements or sensory deficits.  Magnitude not reported. |
| (Jung et al. 2006) | > 2.5 SD above mean of controls | **Upper extremity:** TP = 24/98 limbs, FP = NR, TN = NR, FN = 74/98 limbs  **Lower extremity:** TP = 68/98 limbs, FP = NR, TN = NR, FN = /98 limbs | **Upper extremity:** 25%  **Lower extremity:** 69% (68-70%) | NR | NR | Upper extremity, but not lower extremity, CMCT was correlated with pyramidal dysfunction (hyperreflexia, weakness, spasticity, plantar reflex) (*p* < .005), but not corpus callosum atrophy or MRI lesion volume or number.  Magnitude not reported. |
| (Kale et al. 2009) | > 2.5 SD above mean of controls | **Upper extremity:** TP = 64/131 participants, FP = NR, TN = NR, FN = 67/131 participants | **Upper extremity:** 49% | NR | NR | CMCT was correlated with EDSS (*p* < .001).  Magnitude not reported. |
| (Kale et al. 2010) | > 2.5 SD above mean of controls | **Upper extremity:** TP = 32/79 participants, FP = NR, TN = NR, FN = 47/79 participants | **Upper extremity:** 41% | NR | NR | CMCT was correlated with corpus callosum atrophy (*p* not reported) but not EDSS.  Magnitude not reported. |
| (Kandler et al. 1991) | > upper 99% confidence limit of controls,  Asymmetry > upper 99% confidence limit of controls | **Upper extremity:** TP = 27/63 participants, FP = NR, TN = NR, FN = 36/63 participant  **Lower extremity:** TP = 42/63 participants, FP = NR, TN = NR, FN = 21/63 participants | **Upper extremity:** 43%  **Lower extremity:** 67% (66-68%) | NR | NR | NR |
| (Leocani et al. 2006) | > 2.5 SD above mean of controls,  Asymmetry > 2.5 SD above mean of controls | **RMS (upper extremity):** TP = 24/43 participants, FP = NR, TN = NR, FN = 19/43 participants, **SPMS (upper extremity):** TP = 26/28 participants, FP = NR, TN = NR, FN = 2/28 participants, **PPMS (upper extremity):** TP = 11/13 participants, FP = NR, TN = NR, FN = 2/13 participants, **RMS (lower extremity):** TP = 26/43 participants, FP = NR, TN = NR, FN = 17/43 participants, **SPMS (lower extremity):** TP = 27/28 participants, FP = NR, TN = NR, FN = 1/28 participants, **PPMS (lower extremity):** TP = 12/13 participants, FP = NR, TN = NR, FN = 1/13 participants | **RMS (upper extremity):** 56% (54-58%)  **SPMS (upper extremity):** 93% (87-99%)  **PPMS (upper extremity):** 85% (72-98%)  **RMS (lower extremity):** 61% RMS (59-63%)  **SPMS (lower extremity):** 96% SPMS (89-100%)  **PPMS (lower extremity):** 92% PPMS (78-100%) | NR | NR | CMCT was correlated with EDSS (Rho = 0.6, *p* < .001). |
| (Magistris et al. 1999) | > 2.5 SD above lab normative values | **Upper extremity:** TP = 60/221 limbs, FP = 112/268 limbs, TN = 156/268 limbs, FN = 161/221 limbs | **Upper extremity:** 27% | **Upper extremity:** 58% (58-58%) | **Upper extremity:** 0.52 (0.35-0.76)ꝉ | CMCT was not significantly correlated with weakness. |
| (Mayr et al. 1991) | > 99 %ile of controls | **Upper extremity:** TP = 31/44 participants, FP = 0/86 participants, TN = 86/86 participants, FN = 13/44 participants  **Lower extremity:** TP = 27/44 participants, FP = 0/86 participants, TN = 86/86 participants, FN = 17/44 participants | **Upper extremity:** 71% (68-74%)  **Lower extremity:** 61% (59-63%) | **Upper extremity:** 99% (97-100%)  **Lower extremity:** 100% (98-100%) | **Upper extremity:** 202.69 (102.56-400.59)  **Lower extremity:** 135.00 (71.00-256.69) | CMCT was correlated with pyramidal dysfunction (hyperreflexia, weakness, spasticity, plantar reflex) (*p* not reported).  Magnitude not reported. |
| (Ravnborg et al. 1992) | > upper 99% confidence limit of controls | **Upper + lower extremity:** TP = 33/40 participants, FP = 7/28 participants, TN = 21/28 participants, FN = 7/40 participants | **Upper + lower extremity:** 83% (73-93%) | **Upper + lower extremity:** 75% (61-89%) | **Upper + lower extremity:** 14.14 (4.34-46.11) | CMCT was correlated with MRI lesion number (McNemar’s = 0.85, *p* not reported) but not pyramidal dysfunction (hyperreflexia, weakness, spasticity, plantar reflex). |
| (Schmierer et al. 2000) | > 2.5 SD above mean of controls | **Upper extremity:** TP = 7/50 participants, FP = 0/25 participants, TN = 25/25 participants, FN = 43/50 participants  **Lower extremity:** TP = 24/50 participants, FP = 0/25 participants, TN = 25/25 participants, FN = 26/50 participants | **Upper extremity:** 14%  **Lower extremity:** 48% | **Upper extremity:** 100% (92-100%)  **Lower extremity:** 100% (92-100%) | **Upper extremity:** 3.91 (1.61-9.52)  **Lower extremity:** 22.15 (11.23-43.69) | CMCT was not significantly correlated with MRI lesion burden or location. |
| (Schmierer et al. 2002) | > 2.5 SD above mean of controls | **RMS (upper extremity):** TP = 12/38 limbs, FP = NR, TN = NR, FN = 26/38 limbs  **PPMS (upper extremity):** TP = 14/38 limbs, FP = NR, TN = NR, FN = 24/38 limbs  **RMS (lower extremity):** TP = 24/38 limbs, FP = NR, TN = NR, FN = 14/38 limbs  **PPMS (lower extremity):** TP = 22/38 limbs, FP = NR, TN = NR, FN = 16/38 limbs | **RMS (upper extremity):** 32%  **PPMS (upper extremity):** 37%  **RMS (lower extremity):** 63% (60-66%)  **PPMS (lower extremity):** 58% (56-60%) | NR | NR | Upper and lower extremity CMCT was correlated with EDSS (*r* = 0.4-0.5, *p* < .01). |
| (Tataroglu et al. 2003) | > 2.5 SD above mean of controls | **Upper + lower extremity:** TP = 44/58 participants, FP = 4/31 participants, TN = 27/31 participants, FN = 14/58 participants | **Upper + lower extremity:** 76% (74-78%) | **Upper + lower extremity:** 87% (82-92%) | **Upper + lower extremity:** 21.21 (6.32-71.14) | CMCT was not significantly correlated with EDSS. |
| ***Triple stimulation technique (TST), one study (6%)*** | | | | | | |
| (Magistris et al. 1999) | > 2.5 SD below laboratory normative values | **Upper extremity:** TP = 106/221 limbs, FP = 106/268 limbs, TN = 162/268 limbs, FN = 115/221 limbs, | **Upper extremity:** 48% | **Upper extremity:** 60% (60-60%) | **Upper extremity:** 0.60 (0.42-0.86)ꝉ | TST amplitude ratio was correlated with weakness (*p* < .0001).  Magnitude not reported. |
| ***Corticospinal silent period (CSP), one study (6%)*** | | | | | | |
| (Tataroglu et al. 2003) | > 2.5 SD above mean of controls,  Asymmetry > 2.5 SD above mean of controls | **Upper + lower extremity:** TP = 40/58 participants, FP = 9/31 participants, TN = 22/31 participants, FN = 18/58 participants | **Upper + lower extremity:** 69% (67-71%) | **Upper + lower extremity:** 70% (66-74%) | **Upper + lower extremity:** 5.43 (2.09-14.10) | CSP duration was correlated with ataxia (*r* = 0.3, *p* < .001) but not EDSS. |
| ***Ipsilateral silent period (iSP), three studies (16%)*** | | | | | | |
| *iSP latency, three studies (16%)* | | | | | | |
| (Jung et al. 2006) | > 2.5 SD above mean of controls,  Asymmetry > 2.5 SD above mean of controls | TP = 4/98 limbs, FP = NR, TN = NR, FN = 94/98 limbs | **Upper extremity:** 4% | NR | NR | iSP latency was not significantly correlated with pyramidal dysfunction (hyperreflexia, weakness, spasticity, plantar reflex), corpus callosum atrophy, or MRI lesion volume or number. |
| (Schmierer et al. 2000) | > 2.5 SD above mean of controls | TP = 9/50 participants, FP = 0/25 participants, TN = 25/25 participants, FN = 41/50 participants | **Upper extremity:** 18% | **Upper extremity:** 100% (92-100%) | **Upper extremity:** 5.27 (2.32-11.98) | iSP latency was not significantly correlated with MRI lesion burden or location. |
| (Schmierer et al. 2002) | > 2.5 SD above mean of controls | **RMS:** TP = 6/38 limbs, FP = NR, TN = NR, FN = 32/38 limbs  **PPMS:** TP = 13/38 limbs, FP = NR, TN = NR, FN = 25/38 limbs | **Upper extremity RMS:** 16%  **Upper extremity PPMS:** 34% | NR | NR | iSP latency was correlated with EDSS in PPMS (*r* = 0.4, *p* < .01) but not RMS. |

| *iSP duration, three studies (18%)* | | | | | | |
| --- | --- | --- | --- | --- | --- | --- |
| (Jung et al. 2006) | > 2.5 SD above mean of controls,  Asymmetry > 2.5 SD above mean of controls | TP = 22/98 limbs, FP = NR, TN = NR, FN = 76/98 limbs | **Upper extremity:** 22% | NR | NR | iSP duration was not significantly correlated with pyramidal dysfunction (hyperreflexia, weakness, spasticity, plantar reflex), corpus callosum atrophy, or MRI lesion volume or number. |
| (Schmierer et al. 2000) | > 2.5 SD above mean of controls | TP = 36/50 participants, FP = 0/25 participants, TN = 25/25 participants, FN = 14/50 participants | **Upper extremity:** 72% (69-75%) | **Upper extremity:** 100% (92-100%) | **Upper extremity:** 61.71 (29.70-128.22) | iSP duration was with MRI lesion burden (*r* = 0.4, *p* < .01) but not MRI lesion location. |
| (Schmierer et al. 2002) | > 2.5 SD above mean of controls | **RMS:** TP = 13/38 limbs, FP = NR, TN = NR, FN = 25/38 limbs  **PPMS:** TP = 19/38 limbs, FP = NR, TN = NR, FN = 19/38 limbs | **Upper extremity RMS:** 16%  **Upper extremity PPMS:** 34% | NR | NR | iSP duration was not significantly correlated with EDSS. |
| *iSP depth, one study (6%)* | | | | | | |
| (Jung et al. 2006) | > 2.5 SD above mean of controls | TP = 6/98 limbs, FP = NR, TN = NR, FN = 92/98 limbs | **Upper extremity:** 6% | NR | NR | iSP depth was not significantly correlated with pyramidal dysfunction (hyperreflexia, weakness, spasticity, plantar reflex), corpus callosum atrophy, or MRI lesion volume or number. |
| *Transcallosal conduction time (TCT), three studies (18%)* | | | | | | |
| (Jung et al. 2006) | > 2.5 SD above mean of controls,  Asymmetry > 2.5 SD above mean of controls | TP = 6/98 limbs, FP = NR, TN = NR, FN = 92/98 limbs | **Upper extremity:** 6% | NR | NR | TCT was not significantly correlated with pyramidal dysfunction (hyperreflexia, weakness, spasticity, plantar reflex), corpus callosum atrophy, or MRI lesion volume or number. |
| (Schmierer et al. 2000) | > 2.5 SD above mean of controls | TP = 2/50 participants, FP = 0/25 participants, TN = 25/25 participants, FN = 48/50 participants | **Upper extremity:** 4% | **Upper extremity:** 100% (92-100%) | **Upper extremity:** 1.00 (0.23-4.34)* | TCT was not significantly correlated with MRI lesion burden or location. |
| (Schmierer et al. 2002) | > 2.5 SD above mean of controls | **RMS:** TP = 5/38 limbs, FP = NR, TN = NR, FN = 33/38 limbs  **PPMS:** TP = 9/38 limbs, FP = NR, TN = NR, FN = 29/38 limbs | **Upper extremity RMS:** 13%  **Upper extremity PPMS:** 24% | NR | NR | TCT was not significantly correlated with EDSS. |

%ile, percentile; 95% CI, 95% confidence interval; CMAP, compound muscle action potential; EDSS, Expanded Disability Status Scale; FN, false negative; FP, false positive; MRI, magnetic resonance imaging; NR, not reported; PPMS, primary progressive multiple sclerosis RMS, relapsing multiple sclerosis; SD, standard deviation; SPMS, secondary progressive multiple sclerosis; TN, true negative; TP, true positive. *, 95% CI of diagnostic odds ratio (DOR) crossed zero, suggesting no change in odds of MS. ꝉ, DOR < 1 indicated decreased odds of MS.

**Table S5.** Detailed risk of bias assessment.

|  | **Domain** | **Patient Selection** | **Index Test** | **Reference Standard** | **Flow and Timing** | **Risk of Bias** |
| --- | --- | --- | --- | --- | --- | --- |
| **Article** | Description | Describe methods of patient selection Describe included patients (previous testing, presentation, intended use of index test, and setting) | Describe the index test and how it was conducted and interpreted | Describe the reference standard and how it was conducted and interpreted | Describe any patients who did not receive the index tests or reference standard or who were excluded from the 2 × 2 table (refer to flow diagram) Describe the interval and any interventions between index tests and the reference standard | NA |
|  | Signaling Questions (Y / N / U) | Was a consecutive or random sample of patients enrolled? | Were the index test results interpreted without knowledge of the results of the reference standard? | Is the reference standard likely to correctly classify the target condition? | Was there an appropriate interval between index tests and reference standard? |  |
|  |  | Was a case–control design avoided? |  |  | Did all patients receive a reference standard? |  |
|  |  | Did the study avoid inappropriate exclusions? | If a threshold was used, was it prespecified? | Were the reference standard results interpreted without knowledge of the results of the index test? | Did all patients receive the same reference standard? |  |
|  |  |  |  |  | Were all patients included in the analysis? |  |
|  | Risk of Bias (H / L / U) | Could the selection of patients have introduced bias? | Could the conduct or interpretation of the index test have introduced bias? | Could the reference standard, its conduct, or its interpretation have introduced bias? | Could the patient flow have introduced bias? (Was the patient flow free of bias?) | What was the study's overall risk of bias? |
|  | Concerns About Applicability (H / L / U) | Are there concerns that the included patients do not match the review question? | Are there concerns that the index test, its conduct, or its interpretation differ from the review question? | Are there concerns that the target condition as defined by the reference standard does not match the review question? | NA | Were there concerns for applicability of the overall study methods? |
| (Beer et al. 1995) | Signaling Questions (Y / N / U) | Y | Y | Y | Y | NA |
|  |  | Y |  |  | Y |  |
|  |  | Y | Y | Y | Y |  |
|  |  |  |  |  | Y |  |
|  | Risk of Bias (H / L / U) | L | L | L | L | L |
|  | Concerns About Applicability (H / L / U) | L | L | L | NA | L |
| (Caramia et al. 2004) | Signaling Questions (Y / N / U) | U | N | Y | Y | NA |
|  |  | Y |  |  | Y |  |
|  |  | U | Y | N | N |  |
|  |  |  |  |  | Y |  |
|  | Risk of Bias (H / L / U) | U | H | H | H | H |
|  | Concerns About Applicability (H / L / U) | U | L | L | NA | U |
| (Cruz-Martínez et al. 2000) | Signaling Questions (Y / N / U) | Y | Y | Y | Y | NA |
|  |  | Y |  |  | Y |  |
|  |  | U | Y | Y | Y |  |
|  |  |  |  |  | U |  |
|  | Risk of Bias (H / L / U) | U | L | L | U | U |
|  | Concerns About Applicability (H / L / U) | U | L | L | NA | U |
| (Facchetti et al. 1997) | Signaling Questions (Y / N / U) | Y | Y | Y | Y | NA |
|  |  | N |  |  | Y |  |
|  |  | N | Y | Y | Y |  |
|  |  |  |  |  | Y |  |
|  | Risk of Bias (H / L / U) | H | L | L | L | H |
|  | Concerns About Applicability (H / L / U) | H | L | L | NA | H |
| (Hess et al. 1987) | Signaling Questions (Y / N / U) | N | N | Y | Y | NA |
|  |  | N |  |  | Y |  |
|  |  | Y | Y | N | N |  |
|  |  |  |  |  | Y |  |
|  | Risk of Bias (H / L / U) | H | H | H | H | H |
|  | Concerns About Applicability (H / L / U) | L | L | L | NA | L |
| (Jung et al. 2006) | Signaling Questions (Y / N / U) | U | U | Y | Y | NA |
|  |  | N |  |  | Y |  |
|  |  | U | Y | U | Y |  |
|  |  |  |  |  | Y |  |
|  | Risk of Bias (H / L / U) | H | U | U | L | H |
|  | Concerns About Applicability (H / L / U) | U | L | L | NA | U |
| (Kale et al. 2009) | Signaling Questions (Y / N / U) | Y | U | Y | Y | NA |
|  |  | N |  |  | Y |  |
|  |  | N | Y | U | Y |  |
|  |  |  |  |  | Y |  |
|  | Risk of Bias (H / L / U) | H | U | U | L | H |
|  | Concerns About Applicability (H / L / U) | H | L | L | NA | H |
| (Kale et al. 2010) | Signaling Questions (Y / N / U) | N | U | Y | Y | NA |
|  |  | N |  |  | Y |  |
|  |  | N | Y | U | Y |  |
|  |  |  |  |  | Y |  |
|  | Risk of Bias (H / L / U) | H | U | U | L | H |
|  | Concerns About Applicability (H / L / U) | H | L | L | NA | H |
| (Kandler et al. 1991) | Signaling Questions (Y / N / U) | Y | U | Y | Y | NA |
|  |  | N |  |  | Y |  |
|  |  | Y | Y | U | Y |  |
|  |  |  |  |  | N |  |
|  | Risk of Bias (H / L / U) | H | U | U | H | H |
|  | Concerns About Applicability (H / L / U) | L | L | L | NA | U |
| (Leocani et al. 2006) | Signaling Questions (Y / N / U) | Y | Y | Y | Y | NA |
|  |  | Y |  |  | Y |  |
|  |  | N | Y | Y | Y |  |
|  |  |  |  |  | Y |  |
|  | Risk of Bias (H / L / U) | H | L | L | L | H |
|  | Concerns About Applicability (H / L / U) | H | L | L | NA | H |
| (Magistris et al. 1999) | Signaling Questions (Y / N / U) | Y | U | Y | Y | NA |
|  |  | Y |  |  | Y |  |
|  |  | Y | Y | U | Y |  |
|  |  |  |  |  | Y |  |
|  | Risk of Bias (H / L / U) | L | U | U | L | U |
|  | Concerns About Applicability (H / L / U) | L | L | L | NA | L |
| (Mayr et al. 1991) | Signaling Questions (Y / N / U) | U | U | Y | Y | NA |
|  |  | N |  |  | Y |  |
|  |  | N | Y | U | Y |  |
|  |  |  |  |  | Y |  |
|  | Risk of Bias (H / L / U) | H | U | U | L | H |
|  | Concerns About Applicability (H / L / U) | H | L | L | NA | H |
| (Pisa et al. 2020) | Signaling Questions (Y / N / U) | Y | U | Y | Y | NA |
|  |  | N |  |  | Y |  |
|  |  | Y | Y | U | Y |  |
|  |  |  |  |  | Y |  |
|  | Risk of Bias (H / L / U) | H | U | U | L | H |
|  | Concerns About Applicability (H / L / U) | L | L | L | NA | L |
| (Ravnborg et al. 1992) | Signaling Questions (Y / N / U) | Y | Y | Y | Y | NA |
|  |  | Y |  |  | Y |  |
|  |  | Y | Y | Y | Y |  |
|  |  |  |  |  | Y |  |
|  | Risk of Bias (H / L / U) | L | L | L | L | L |
|  | Concerns About Applicability (H / L / U) | L | L | L | NA | L |
| (Schmierer et al. 2000) | Signaling Questions (Y / N / U) | U | U | Y | Y | NA |
|  |  | N |  |  | Y |  |
|  |  | N | Y | U | Y |  |
|  |  |  |  |  | Y |  |
|  | Risk of Bias (H / L / U) | H | U | U | L | H |
|  | Concerns About Applicability (H / L / U) | H | L | L | NA | H |
| (Schmierer et al. 2002) | Signaling Questions (Y / N / U) | U | U | Y | Y | NA |
|  |  | Y |  |  | Y |  |
|  |  | N | Y | U | Y |  |
|  |  |  |  |  | N |  |
|  | Risk of Bias (H / L / U) | H | U | U | H | H |
|  | Concerns About Applicability (H / L / U) | H | L | L | NA | H |
| (Tataroglu et al. 2003) | Signaling Questions (Y / N / U) | Y | U | Y | Y | NA |
|  |  | N |  |  | Y |  |
|  |  | N | Y | U | Y |  |
|  |  |  |  |  | U |  |
|  | Risk of Bias (H / L / U) | H | U | U | U | H |
|  | Concerns About Applicability (H / L / U) | H | L | L | NA | H |

H, high; L, low; N, no; NA, not applicable; U, unclear; Y, yes. See ref: (Whiting et al. 2011).

**Table S6.** Detailed biomarker assessment.

| **Study** | **Process-specific classification** | | | | | | | **Methodologic quality** | | | | | **Clinical utility** | | | | | **Clinical usefulness** | | | | **Biomarker validity** |
| --- | --- | --- | --- | --- | --- | --- | --- | --- | --- | --- | --- | --- | --- | --- | --- | --- | --- | --- | --- | --- | --- | --- |
|  | **Immune alteration** | **BBB disruption** | **Demyelination** | **Excitotoxicity** | **Axonal damage** | **Gliosis** | **Remyelination** | **Raw data** | **Reference standard** | **Appropriate patient spectrum** | **Valid methods** | **Safeguards against bias** | **Biological rationale** | **Clinical relevance** | **Practicality** | **Correlation with disease** | **Correlation with disability** | **Sensitivity** | **Specificity** | **Reliability** | **Epidemiologic evaluation** |  |
| (Beer et al. 1995) | U | U | U | U | U | U | U | N | Y | Y | Y | Y | U | Y | U | U | U | N | N | U | N | N |
| (Caramia et al. 2004) | U | U | Y | U | Y | U | N | N | Y | U | Y | N | Y | U | U | N | U | N | U | Y | N | N |
| (Cruz-Martínez et al. 2000) | U | U | Y | U | Y | U | U | N | Y | Y | Y | Y | Y | Y | Y | Y | Y | N | Y | U | N | N |
| (Facchetti et al. 1997) | U | U | Y | U | Y | U | U | N | Y | Y | U | Y | Y | Y | Y | Y | U | Y | U | U | U | N |
| (Hess et al. 1987) | U | U | Y | U | Y | U | U | N | Y | Y | Y | N | Y | Y | Y | U | U | N | Y | Y | N | N |
| (Jung et al. 2006) | U | U | Y | U | Y | U | U | N | Y | U | Y | U | Y | N | N | N | U | N | U | U | N | N |
| (Kale et al. 2009) | U | U | Y | U | Y | U | U | N | Y | Y | U | U | Y | Y | Y | U | Y | N | U | U | N | N |
| (Kale et al. 2010) | U | U | Y | U | Y | U | U | N | Y | U | U | U | Y | U | U | U | Y | Y | U | U | U | N |
| (Kandler et al. 1991) | U | U | Y | U | U | U | U | N | Y | Y | Y | U | Y | U | U | U | U | N | U | U | N | N |
| (Leocani et al. 2006) | U | U | Y | U | Y | U | Y | N | Y | Y | U | Y | Y | Y | Y | U | Y | Y | U | Y | U | N |
| (Magistris et al. 1999) | U | U | Y | U | Y | U | U | N | Y | Y | Y | U | Y | Y | Y | U | U | N | N | Y | N | N |
| (Mayr et al. 1991) | U | U | Y | U | U | U | U | Y | Y | U | Y | U | Y | U | Y | U | U | N | Y | U | N | N |
| (Pisa et al. 2020) | U | U | Y | U | U | U | U | N | Y | Y | Y | U | Y | U | U | U | Y | Y | U | U | U | N |
| (Ravnborg et al. 1992) | U | U | Y | U | U | U | U | N | Y | Y | Y | Y | Y | U | U | Y | N | N | Y | U | N | N |
| (Schmierer et al. 2000) | U | U | Y | U | Y | U | U | N | Y | U | Y | U | Y | Y | Y | Y | U | N | Y | U | N | N |
| (Schmierer et al. 2002) | U | U | Y | U | Y | U | U | N | Y | U | Y | U | Y | U | U | U | Y | N | U | Y | N | N |
| (Tataroglu et al. 2003) | U | U | Y | Y | Y | U | U | N | Y | Y | U | U | Y | Y | Y | U | Y | N | Y | U | N | N |

Note: criteria related to treatment efficacy are omitted. BBB, blood-brain barrier; H, high; L, low; N, no; U, uncertain. See ref: (Bielekova & Martin 2004).

**References**

Beer S, Rösler KM, and Hess CW. 1995. Diagnostic value of paraclinical tests in multiple sclerosis: relative sensitivities and specificities for reclassification according to the Poser committee criteria. *Journal of Neurology, Neurosurgery, and Psychiatry* 59:152–159. 10.1136/jnnp.59.2.152

Bielekova B, and Martin R. 2004. Development of biomarkers in multiple sclerosis. *Brain* 127:1463-1478. 10.1093/brain/awh176

Caramia MD, Palmieri MG, Desiato MT, Boffa L, Galizia P, Rossini PM, Centonze D, and Bernardi G. 2004. Brain excitability changes in the relapsing and remitting phases of multiple sclerosis: a study with transcranial magnetic stimulation. *Clin Neurophysiol* 115:956-965. 10.1016/j.clinph.2003.11.024

Chipchase L, Schabrun S, Cohen L, Hodges P, Ridding M, Rothwell J, Taylor J, and Ziemann U. 2012. A checklist for assessing the methodological quality of studies using transcranial magnetic stimulation to study the motor system: an international consensus study. *Clin Neurophysiol* 123:1698-1704. 10.1016/j.clinph.2012.05.003

Cruz-Martínez A, Gonzalez-Orodea JI, Lopez Pajares R, and Arpa J. 2000. Disability in Multiple Sclerosis. The role of Transcranial Magnetic Stimulation. *Electroencephalography and Clinical Neurophysiology* 40:441-447.

Facchetti D, Mai R, Micheli A, Marciano N, Capra R, Gasparotti R, and Poloni M. 1997. Motor evoked potentials and disability in secondary progressive multiple sclerosis. *Can J Neurol Sci* 24:332-337. 10.1017/s0317167100033011

Hess CW, Mills KR, Murray NMF, and Schriefer TN. 1987. Magnetic Brain Stimulation: Central Motor Conduction Studies in Multiple Sclerosis. *Annals of Neurology* 22:744-752.

Jung P, Beyerle A, Humpich M, Neumann-Haefelin T, Lanfermann H, and Ziemann U. 2006. Ipsilateral silent period: a marker of callosal conduction abnormality in early relapsing-remitting multiple sclerosis? *J Neurol Sci* 250:133-139. 10.1016/j.jns.2006.08.008

Kale N, Agaoglu J, Onder G, and Tanik O. 2009. Correlation between disability and transcranial magnetic stimulation abnormalities in patients with multiple sclerosis. *J Clin Neurosci* 16:1439-1442. 10.1016/j.jocn.2009.03.009

Kale N, Agaoglu J, and Tanik O. 2010. Electrophysiological and clinical correlates of corpus callosum atrophy in patients with multiple sclerosis. *Neurol Res* 32:886-890. 10.1179/016164109x12445616596526

Kandler RH, Jarratt JA, Gumpert EJW, Davies-Jones GAB, Venables GS, and Sagar HJ. 1991. The role of magnetic stimulation in the diagnosis of multiple sclerosis. *Journal of the Neurological Sciences* 106:25-30.

Leocani L, Rovaris M, Boneschi FM, Medaglini S, Rossi P, Martinelli V, Amadio S, and Comi G. 2006. Multimodal evoked potentials to assess the evolution of multiple sclerosis: a longitudinal study. *J Neurol Neurosurg Psychiatry* 77:1030-1035. 10.1136/jnnp.2005.086280

Magistris MR, Rosler KM, Truffert A, Landis T, and Hess CW. 1999. A clinical study of motor evoked potentials using a triple stimulation technique. *Brain* 122:265-279.

Mayr N, Baumgartner C, Zeitlhofer J, and Deecke L. 1991. The sensitivity of transcranial cortical magnetic stimulation in detecting pyramidal tract lesions in clinically definite multiple sclerosis. *Neurology* 41:566-569.

Pisa M, Chieffo R, Giordano A, Gelibter S, Comola M, Comi G, and Leocani L. 2020. Upper limb motor evoked potentials as outcome measure in progressive multiple sclerosis. *Clin Neurophysiol* 131:401-405. 10.1016/j.clinph.2019.11.024

Ravnborg M, Liguori R, Christiansen P, Larsson H, and Sorensen PS. 1992. The diagnostic reliability of magnetically evoked motor potentials in multiple sclerosis. *Neurology* 42:1296-1301.

Schmierer K, Irlbacher K, Grosse P, Röricht S, and Meyer BU. 2002. Correlates of disability in multiple sclerosis detected by transcranial magnetic stimulation. *Neurology* 59:1218-1224.

Schmierer K, Niehaus L, Röricht S, and Meyer BU. 2000. Conduction deficits of callosal fibres in early multiple sclerosis. *Journal of Neurology, Neurosurgery & Psychiatry* 68:633-638.

Tataroglu C, Genc A, Idiman E, Cakmur R, and Idiman F. 2003. Cortical silent period and motor evoked potentials in patients with multiple sclerosis. *Clinical Neurology and Neurosurgery* 105:105-110. 10.1016/s0303-8467(02)00127-0

Whiting PF, Rutjes AWS, Westwood ME, Mallett S, Deeks JJ, Reitsma JB, Leeflang MMG, Sterne JAC, Bossuyt PMM, and Group TQ-. 2011. QUADAS-2: A Revised Tool for the Quality Assessment of Diagnostic Accuracy Studies. *Annals of Internal Medicine* 155:529-536.
